# Boric Acid Solution Inhibits *Candida albicans* Infections in Mouse Skin *via* the IL-23/Th17 Axis

**DOI:** 10.3389/fmicb.2022.919677

**Published:** 2022-06-16

**Authors:** Zhao Liu, Qing Liu, Yanyan Xu, Zhao Han, Ling Zhang, Xiaojing Li

**Affiliations:** ^1^Department of Dermatology, Affiliated Hospital of Hebei University of Engineering, Handan, China; ^2^Department of Dermatology, Anqing Municipal Hospital, Anqing, China

**Keywords:** mice, *Candida albicans*, interleukin-17, interleukin-22, interleukin-23, 3% boric acid solution

## Abstract

The purpose of this study was to investigate the effect and mechanism of 3% boric acid solution (BAS) against *Candida albicans* (CA) infection *via* the interleukin-23 (IL-23)/T helper 17 cell (Th17) axis. 36 female mice were randomly divided into 3 groups, and 2 injection sites on the back of the mice were chosen at random. Group N was injected with sterile water for injection (SWFI), and Group M and Group B were injected with CA mycelium suspension. After successful model verification, the remaining mice entered the following treatments 5 days later. Group B was treated with 3% BAS, Group M was treated with SWFI, and Group N was not treated. Levels of interleukin-17 (IL-17), IL-22, and IL-23 in mouse blood were measured on days 1, 3, 5, and 7 of treatment. On day 7, IL-17, IL-22, and IL-23 in mouse skin were detected. Serum levels of IL-17, IL-22, and IL-23 in Group M were higher than in Group N on the first day of treatment (*p* < 0.05). Expression levels of IL-17, IL-22, and IL-23 in the epidermis of the skin lesions in Group M were higher than in Group N on day 7 (*p* < 0.05). The serum level of IL-17 in Group B was higher than in Group M on days 5 and 7 (*p* < 0.05). Serum levels of IL-22 in Group B on days 1, 5, and 7 were higher than in Group M (*p* < 0.05). Serum levels of IL-23 in Group B were higher than in Group M on days 3, 5, and 7 (*p* < 0.05). IL-17 and IL-23 in Group B reached a peak on day 5, significantly different on days 1, 3, and 7 (*p* < 0.05). The expression intensity of IL-17, IL-22, and IL-23 in the skin lesions of Group B was higher than that of Group M on day 7 (*p* < 0.05). We conclude that IL-17, IL-22, and IL-23 are involved in the anti-CA activity in mouse skin, and 3% BAS increased IL-17, IL-22, and IL-23 to mediate these effects.

## Introduction

*Candida albicans* (CA) can cause infections on the skin or mucous membranes and invasive infections. Epidemiological studies in the United States and Europe have shown that CA is the most common *Candida* infection (Cleveland et al., 2015; Klingspor et al., 2015; Polesello et al., 2017). It can exist in the human skin, genitourinary tract, and intestines. It is a conditional pathogenic fungus, usually related to the decline of the host's immunity or the imbalance of the competitive flora (Berman, 2012). CA infection can often be caused when the superficial competition flora of the human skin is not balanced, or the body's immunity is weakened. As for immunosuppressed patients, *Candida* causes severe deep infection, with a mortality rate of 46–75% (Brown et al., 2012).

Superficial skin CA infections are usually treated with topical ointments containing azole drugs and allylamine drugs. Deep CA infection requires an oral or intravenous infusion of antifungal drugs, such as polyenes, azoles, allylamines, and echinocandins. Due to the side effects of antifungal drugs and the increased resistance of CA antifungal drugs (Xiao et al., 2018), it is necessary to find a safe and effective alternative therapy. BAS is a commonly used topical medicine in dermatology. When women have failed conventional treatment due to intravaginal Candida infection, boric acid is a safe, alternative, and economical choice for women with recurrent symptoms of chronic vaginitis, and there is no interaction with common antifungal agents (Iavazzo et al., 2011; Schmidt et al., 2018).

Human innate immunity and adaptive immune system have essential functions in resisting CA infection, among which the IL-23/Th17 axis plays an important role. Naive CD4+ T cells differentiate into various T cell subpopulations, such as Th17 cells, which secrete IL-17 and IL-22. IL-17 promotes the secretion of chemokines by epithelial cells and eliminates fungi by attracting and activating neutrophils (Yang et al., 2015). IL-22 combats fungi in keratinocytes by assisting the production of antibacterial peptides and inflammatory factors (Yang et al., 2015). Active dendritic cells and macrophages secrete IL-23, which promotes the increase in the number of Th17 cells and promotes the production of IL-17 and IL-22 (Ge et al., 2019). Our previous studies showed that 3% boric acid solution (BAS) treats CA infections in mouse skin (Liu et al., 2021); however, the specific mechanism of action has not been thoroughly studied. Therefore, we hypothesized whether BAS increased IL-17, IL-22, and IL-23 in mice, helping the host resist CA infection.

Therefore, we established a mouse skin CA infection model using 3%BAS, based on the IL-23/Th17 axis, to study its mechanism of action against CA infections in mouse skin.

## Materials and Methods

### Laboratory Animals and Strains

Healthy female ICR mice aged 6–8 weeks, weighing 22–24 g, were purchased from the Beijing Weitong Lihua Experimental Animal Technology Co., Ltd. CA standard strain SC 5314 was purchased from the American Type Culture Collection.

### Instruments

BAS the Affiliated Hospital of Hebei Engineering University provided the 3% BAS. IL-17, IL-22, IL-23 Enzyme-linked immunosorbent assay (ELISA) kits were purchased from Jiangsu Meimian Industrial Co., Ltd. IL-17, IL-22, and IL-23 rabbit anti-mouse polyclonal antibodies were purchased from Beijing Biosynthesis Biotechnology Co., Ltd.

### Model Construction and Group Intervention

The mice were randomly divided into N *(n* = 12), M (*n* = 12), and B groups (*n* = 12). The 2 injection sites were randomly selected on the back of each mouse. Groups M and B were injected with CA mycelium suspension and Group N was injected with SWFI. From each group, we randomly selected 6 animals for model verification. Please refer to our published articles for details about the CA mycelium suspension configuration, model establishment, and verification method (Liu et al., 2021).

On day 5 after inoculation, anesthetized mice underwent an intramuscular injection of 0.2 ml chlorpromazine solution. Each mouse in Group B was hydropathic compressed with 6 layers of sterile gauze and 3% BAS for 30 min, once every 12 h. Group M was treated with SWFI, and Group N was fed normally without treatment.

### Sample Collection

On days 1, 3, 5, and 7 of treatment, about 100–120 μL of tail vein blood was collected from each mouse, centrifuged at 1,000 r/min for 10 min, and the upper serum was collected and placed in a refrigerator at −70°C for later use.

After blood samples were collected on day 7, the mice were sacrificed, and tissues were cut from the skin lesions on the back of each mouse, soaked and fixed in formalin solution, and made into wax blocks.

### ELISA

Expression levels of IL-17, IL-22, and IL-23 in mouse serum were measured using ELISA kits. Briefly, serum was brought to room temperature, and we performed the assay strictly according to the manufacturer's instructions to measure the expression levels of IL-17, IL-22, and IL-23 in mouse serum. Data were expressed as pg/mL.

### Immunohistochemistry

First, specimens were cut into 4-μm sections, placed on anti-dropping glass slides, and treated with xylene dewaxing, ethanol hydration, and 1% methanol hydrogen peroxide solution in a microwave oven. Second, we added normal goat serum blocking solution and removed excess liquid after 20 min. Third, we added IL-17 rabbit anti-mouse polyclonal antibody drop wise, placed it in a humidification box overnight at 4°C, and then washed it with phosphate-buffered saline (PBS). Fourth, we added an appropriate amount of biotinylated secondary antibody, placed samples in a 37°C incubator for 20 min, and washed them with PBS. Fifth, we added streptavidin-horseradish peroxidase, placed it in a 37°C incubator for 20 min, and washed it with PBS. Finally, after hematoxylin counterstaining, we used a DAB kit to develop the color, 1% hydrochloric acid ethanol differentiation, 1% amine water inverse blue, ethanol dehydration, and xylene clarification. We used neutral resin to mount the slides and observe using a microscope.

The epidermis of mouse skin is the subject of our study. The criterion is to score the percentage of positive cells under the microscope and the staining intensity (Ren, 2020). Colorless gets 0 points, light yellow gets 1 point, yellowish-brown gets 2 points, and brown gets 3 points. The number of positive staining cells was calculated as follows. We randomly observed 5 high-power microscope fields (400 ×) for each slice and calculated the percentage of positive cells. Positive cells <5% was 0 points, 5–25% was 1 point, 26–50% was 2 points, 51–75% was 3 points, and 76–100% was 4 points. The scores of the 2 were multiplied to provide the positivity grade: 0 was negative (–), 1–4 as weakly positive (+), 5–8 was positive (++), and 9–12 was strongly positive (+++).

### Statistical Analysis

We used SPSS24.0 statistical software to analyze the data. The measurement data of the ELISA results were expressed as the mean ± standard deviation. Repeated measures analysis of variance were used for overall comparison, analysis of variance was used for overall comparisons between 3 groups at the same time point, the least-squares difference was used for pair wise comparison between groups, and a *post-hoc* least-squares difference *t*-test was used for pair-wise comparison of the same group at different time points. The immunohistochemistry results were compared with the overall difference between groups using the independent sample rank-sum test.

## Results

### CA Infection Increases Expression Levels of IL-17, IL-22, and IL-23 in Mice

**Figure 1:**
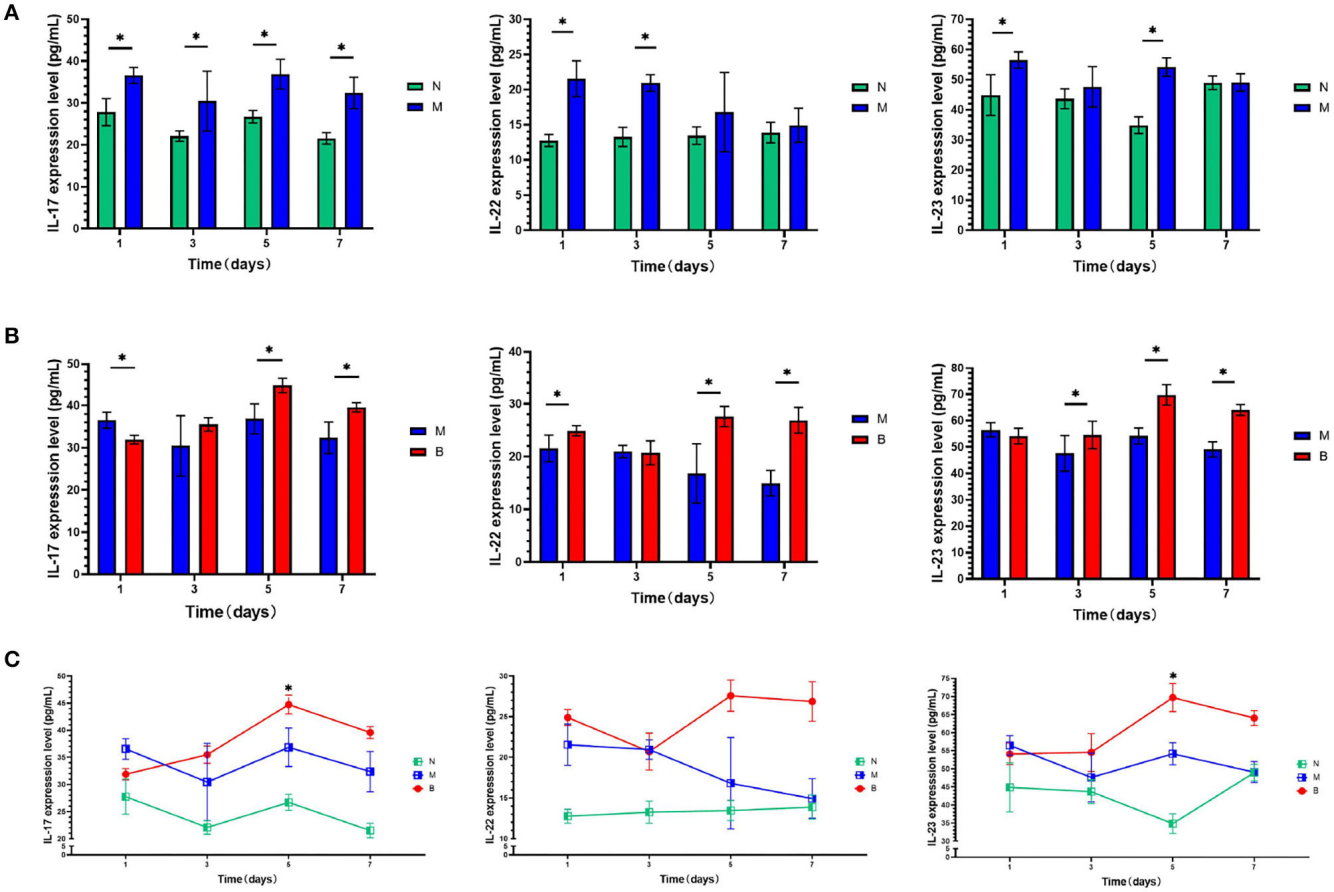
**(A)** On days 1,3, 5, and 7, serum levels of cytokines of the blank group (N) and control group (M) were compared. **(B)** On days 1, 3, 5, and 7, serum levels of cytokines of the control group (M) and the experimental group (B) were compared. **(C)** On days 1,3, 5, and 7, serum levels of cytokines in the same group were compared.*P < 0.05.

**Figure 2:**
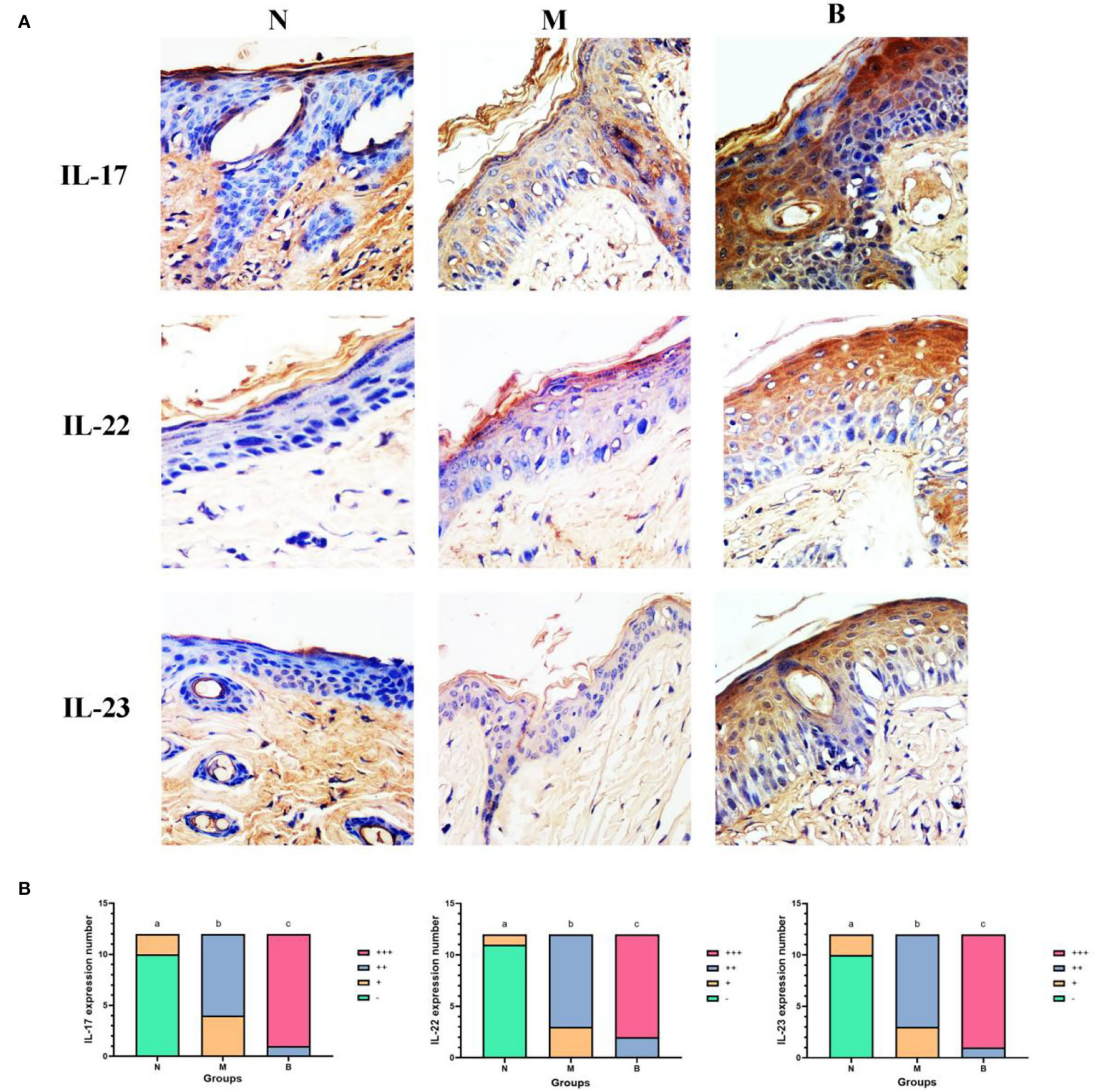
**(A)** The expression of IL-17, IL-22, and IL-23 in the epidermis of each group. The positive color of these 3 cytokines is the coloring of the cytoplasm (light yellow to brown). Negative means no color development. These 3 factors are expressed in different degrees in keratinocytes, especially in spinous cells (400 ×). **(B)** The expression level of IL-17, IL-22, and IL-23 in the epidermis of each group of mice on day 7. Different lowercase letters between groups represent statistically significant differences. *p* < 0.05.

To determine whether CA infection increases expression levels of IL-17, IL-22, and IL-23 in the blood and skin of mice, we compared the M and N groups. On the first day of treatment with a 3% BAS, we performed an ELISA test and found that, as shown in Figure 1A, the expression of IL-17, IL-22, and IL-23 in the blood of mice injected with CA suspension was significantly higher than that of mice injected with SWFI. To avoid the pathological biopsy interfering with the experiment, we did not perform immunohistochemistry on the first day of treatment. On day 7, we performed immunohistochemical testing. The results are shown in Tables 1–3, and Figure 2. IL-17 was negative in ten cases, weakly positive in two cases in the N group, weakly positive in four cases, and positive in eight cases in the M group. IL-22 was negative in 11 cases, weakly positive in one case in Group N, weakly positive in three cases, and positive in nine cases in Group M. IL-23 was negative in 11 cases, weakly positive in one case in Group N, weakly positive in three cases, and positive in nine cases in Group M. These differences are statistically significant. This result confirmed that to resist the infection of CA, the mice secreted more IL-17, IL-22, and IL-23 in blood and skin.

**Table 1:** Comparison of IL-17 expression in three groups of mice epidermis.

**Group**	**Negative**	**Weakly positive**	**Positive**	**Strongly positive**	***Z***	***p***
N (a)	10	2	0	0	31.627	<0.001
M (b)	0	4	8	0		
B (c)	0	0	1	11		

*Different lowercase letters between groups represent statistically significant differences. p < 0.05*.

**Table 2:** Comparison of IL-22 expression in three groups of mice epidermis.

**Group**	**Negative**	**Weakly positive**	**Positive**	**Strongly positive**	***Z***	***p***
N (a)	11	1	0	0	31.46	<0.001
M (b)	0	3	9	0		
B (c)	0	0	2	10		

*Different lowercase letters between groups represent statistically significant differences. p < 0.05*.

**Table 3:** Comparison of IL-23 expression in three groups of mice epidermis.

**Group**	**Negative**	**Weakly positive**	**Positive**	**Strongly positive**	***Z***	***p***
N (a)	10	2	0	0	31.876	<0.001
M (b)	0	3	9	0		
B (c)	0	0	1	11		

*Different lowercase letters between groups represent statistically significant differences. P < 0.05*.

### 3% BAS Increases Expression Levels of IL-17, IL-22, and IL-23 in Mice

To compare whether treatment with 3% BAS would increase the expression levels of IL-17, IL-22, and IL-23 in the blood and skin of mice infected with CA, Groups B and M were compared. As shown in Figure 1B, on day 1, IL-17 expression in the blood of 3% boric acid-treated mice was lower than that of SWFI-treated mice, while on days 5 and 7, IL-17 expression in 3% boric acid-treated mice was significantly higher than in SWFI-treated mice. On days 1, 5, and 7, the expression of IL-22 in the blood of mice treated with 3% boric acid was significantly higher than that of mice treated with SWFI. On days 3, 5, and 7, the expression of IL-23 in the blood of mice treated with 3% boric acid was significantly higher than that of mice treated with SWFI. The results are in Tables 1–3 and Figure 2. IL-17 was positive in one case and strongly positive in 11 cases in Group B, weakly positive in four cases and positive in eight cases in Group M. IL-22 was positive in two cases and strongly positive in ten cases in Group B, and weakly positive in three cases and positive in nine cases in Group M. IL-23 was positive in one case and strongly positive in 11 cases in Group B, and weakly positive in three cases and positive in nine cases in Group M. The expression of these three cytokines in Group B was significantly higher than in Group M. Levels of IL-17, IL-22, and IL-23 in blood and skin were significantly increased after 3% BAS application, which cause may be the result of the breakdown of CA by 3% BAS inhibition of glycolysis and mitochondrial activity (Schmidt et al., 2018), suggesting that the agent helps combat CA infections. Mice treated with 3% BAS were compared at different time points. As shown in Figure 1C, we found that serum levels of IL-17 and IL-23 in mice treated with 3% boric acid on day 5 were significantly higher than at other time points, reaching a peak. However, this phenomenon was not observed in the IL-22 and control groups. These findings suggest that on day 5, IL-17 and IL-23 reached peak levels, and the anti-CA effect was the strongest.

## Discussion

CA is a common conditional pathogenic fungus in humans. It invades tissues, skin, and mucous membranes to cause disease, and then triggers systemic or local inflammatory reactions. Among *Candida* species, CA is the most common disease-causing species, accounting for 75% of *Candida* infections; it is also the most pathogenic (Netea et al., 2015; Dadar et al., 2018).

The host's innate immune response and adaptive immune response are critical for combating CA infections, and the IL-23/Th17 axis plays a vital role. Activated dendritic cells are the primary cells that secrete IL-23; however, they can also be secreted in small amounts by monocytes, macrophages, and keratinocytes (Oppmann et al., 2000; Piskin et al., 2006). IL-23 induces Th17 cells to secrete IL-17 and IL-22 (Bettelli et al., 2007), increases the number of differentiated Th17 cells, and maintains the survival of Th17 cells (Wu et al., 2012). Th17 cells are differentiated from CD4+ helper T cells, which secrete IL-17, IL-22, and other cytokines (Langrish et al., 2005; Park et al., 2005; Fujimura et al., 2013) and play an essential role in the host's resistance to *Candida* infection (Park et al., 2018; Gaffen and Moutsopoulos, 2020). Many studies showed that the secretion of IL-17, IL-22, and IL-23 can help the host resist *Candida* infections, and their absence can cause severe candidiasis (Eyerich et al., 2008; Conti et al., 2009; Kagami et al., 2010). In superficial CA disease, the epidermis is the first barrier to CA infection. Keratinocytes play many roles in resisting CA infection, 1 of which is to secrete antimicrobial peptides. Increased IL-17 and IL-22 secretion promotes the secretion of antimicrobial peptides by keratinocytes, thereby helping the host resist CA infection. We injected CA mycelium suspension and SWFI into the skin of mice and found that serum IL-17, IL-22, and IL-23 in serum were significantly increased on day 1 after CA infection. On day 7, IL-17, IL-22, and IL-23 in mouse skin with CA solution were higher than those injected with SWFI. IL-17 levels increased only in serum. We hypothesized that this phenomenon might be related to decreased cytokine secretion as the infection of CA in the skin is controlled; however, the inflammatory response in the local skin remains strong.

BAS is commonly used in dermatology. Studies showed that boric acid has the same efficacy as fluconazole for treating vaginal candidiasis (Khameneie et al., 2013). Boric acid is a broad-spectrum agent that specifically inhibits CA hyphal growth (Pointer and Schmidt, 2016), and it is more available and less expensive. Our previous study showed that skin CA infection in mice significantly improved after 3% BAS treatment, and there was evident wound healing without exudate. The effective rate of 3% BAS was 83%, and that of SWFI was 25%, which may be caused by discrepancies in drug treatment, operation during treatment, or other factors. Compared with SWFI, 3% BAS had a therapeutic effect and was statistically significant (Liu et al., 2021). However, the mechanism is not entirely clear. Investigators found that BAS destroys the cytoskeleton involving actin and leads to abnormal mycelia development (Pointer et al., 2015). Some investigators found that BAS inhibits glycolysis in CA and critical enzymes of mitochondrial activity (Schmidt et al., 2018). To determine whether 3% BAS stimulates the secretion of IL-17, IL-22, and IL-23, we used 3% BAS and SWFI to treat mice infected with CA by skin injection and found that 3% BAS increased levels of IL-17, IL-22, and IL-23. The expression level of IL-22 in blood was higher than that of the control group on the first day, and the expression level of IL-23 was higher than that of the control group on the third day, while the expression level of IL-17 was higher than that of the control group until the application of 3% BAS on the fifth day. With the application of 3% BAS, the secretion of all 3 cytokines increased in mice.

On day 7, we compared the immunohistochemical tests of 3% BAS and SWFI-treated mice and found that the results were consistent with serum levels of IL-17, IL-22, and IL-23. The expression intensity of IL-17, IL-22, and IL-23 in mice treated with 3% BAS was higher than that of mice treated with SWFI. Previous studies showed that IL-17, IL-22, and IL-23 could help resist *Candida* infections (Eyerich et al., 2008; Conti et al., 2009; Kagami et al., 2010). In addition to the fact that some scholars found that 3% BAS itself had an inhibitory effect on CA (Pointer et al., 2015; Schmidt et al., 2018), we found that it increased IL-17, IL-22, and IL-23 in mice, which cause may be the result of the breakdown of CA by inhibition of glycolysis and mitochondrial activity, to help the host to resist CA infection.

We compared the mice treated with 3% BAS and found that expression levels of IL-17, IL-22, and IL-23 in the blood of mice showed different changes with the extension of application time. The expression peaks of IL-17 and IL-23 appeared on day 5 after treatment with 3% BAS; however, this phenomenon was not observed in IL-22. It usually takes more than 96 h for the antigen recognition to affect the stage of the adaptive immune response, and the time of IL-17 and IL-23 secretion induced by 3% BAS in mice is consistent. Therefore, we speculated that BAS might be involved in a particular stage of the adaptive immune response. The specific mechanism needs to be further explored in the future. Nevertheless, IL-17 and IL-23 secretion peaked on day 5, and its anti-CA effect was also the highest.

In summary, 3% BAS increased IL-17, IL-22, and IL-23 in mice to assist the host in fighting CA infection. To treat CA infections in the skin, the course of treatment should be applied for at least 5 days to achieve the best anti-CA effect.

## Data Availability Statement

The original contributions presented in the study are included in the article/supplementary material, further inquiries can be directed to the corresponding authors.

## Ethics Statement

The animal study was reviewed and approved by The Biomedical Ethics Committee of Medical School of Hebei University of Engineering.

## Author Contributions

ZL wrote the manuscript. XL revised the manuscript. QL, YX, ZH, and LZ gave some helpful suggestions. All authors contributed to manuscript revision, read, and approved the submitted version.

## Conflict of Interest

The authors declare that the research was conducted in the absence of any commercial or financial relationships that could be construed as a potential conflict of interest.

## Publisher's Note

All claims expressed in this article are solely those of the authors and do not necessarily represent those of their affiliated organizations, or those of the publisher, the editors and the reviewers. Any product that may be evaluated in this article, or claim that may be made by its manufacturer, is not guaranteed or endorsed by the publisher.
